# Scaling computational genomics to millions of individuals with GPUs

**DOI:** 10.1186/s13059-019-1836-7

**Published:** 2019-11-01

**Authors:** Amaro Taylor-Weiner, François Aguet, Nicholas J. Haradhvala, Sager Gosai, Shankara Anand, Jaegil Kim, Kristin Ardlie, Eliezer M. Van Allen, Gad Getz

**Affiliations:** 1 Broad Institute of MIT and Harvard, Cambridge, MA USA; 2 Harvard University, Cambridge, MA USA; 3 Department of Medical Oncology, Dana-Farber Cancer Institute, Boston, MA USA; 4 Department of Pathology, Harvard Medical School, Boston, MA USA; 5 Department of Medicine, Harvard Medical School, Boston, MA USA; 6 Cancer Center and Department of Pathology, Massachusetts General Hospital, Boston, MA USA

## Abstract

Current genomics methods are designed to handle tens to thousands of samples but will need to scale to millions to match the pace of data and hypothesis generation in biomedical science. Here, we show that high efficiency at low cost can be achieved by leveraging general-purpose libraries for computing using graphics processing units (GPUs), such as PyTorch and TensorFlow. We demonstrate > 200-fold decreases in runtime and ~ 5–10-fold reductions in cost relative to CPUs. We anticipate that the accessibility of these libraries will lead to a widespread adoption of GPUs in computational genomics.

## Background

Current methodologies for analyzing genomic data were designed for datasets with tens to thousands of samples, but due to the continuing decrease in sequencing costs and growth of large-scale genomic projects, datasets are reaching sizes of millions of samples or single cells. The need for increased computational resources, most notably runtime, to process these growing datasets will become prohibitive without improving the computational efficiency and scalability of methods. For example, methods in population genetics, such as genome-wide association studies (GWAS) or mapping of quantitative trait loci (QTL), involve billions of regressions between genotypes and phenotypes. Currently, the state-of-the-art infrastructures for performing these tasks are large-scale clusters of central processing units (CPUs), often with thousands of cores, resulting in significant costs [1] (960 cores on a standard Google Cloud machine currently costs $7660.80 per day of compute). In contrast to CPUs, a single graphics processing unit (GPU) contains thousands of cores at a much lower price per core (Nvidia’s P100 has 3584 cores and currently costs $35.04 per day of compute).

Previous work has already demonstrated the benefits of using GPUs to scale bioinformatics methods [2–6]. However, these implementations were often complex and based on specialized libraries, limiting their extensibility and adoption. In contrast, recent open-source libraries such as TensorFlow [7] or PyTorch [8], which were developed for machine learning applications but implement general-purpose mathematical primitives and methods (e.g., matrix multiplication), make the development of GPU-compatible tools widely accessible to the research community. These libraries offer several major advantages: (i) they implement most of the functionalities of CPU-based scientific computing libraries such as NumPy, and thus are easy to use for implementing various algorithms; (ii) they easily handle data transfer from the computer’s memory to the GPU’s internal memory, including in batches, and thus greatly facilitate computations on large datasets (e.g., large genotype matrices) that do not fit into the GPU’s memory; (iii) they are trivial to install and run, enabling easy sharing of methods; and (iv) they can run seamlessly on both CPUs and GPUs, permitting users without access to GPUs to test and use them, without loss of performance compared with other CPU-based implementations (Additional file 1: Figure S1). Moreover, users do not need to explicitly specify how to parallelize algorithms across the GPU cores. We hypothesized that the use of these libraries would result in significant improvements in computational efficiency and enable scaling computational genomics methods to millions of samples.

## Results and discussion

**Fig. 1:**
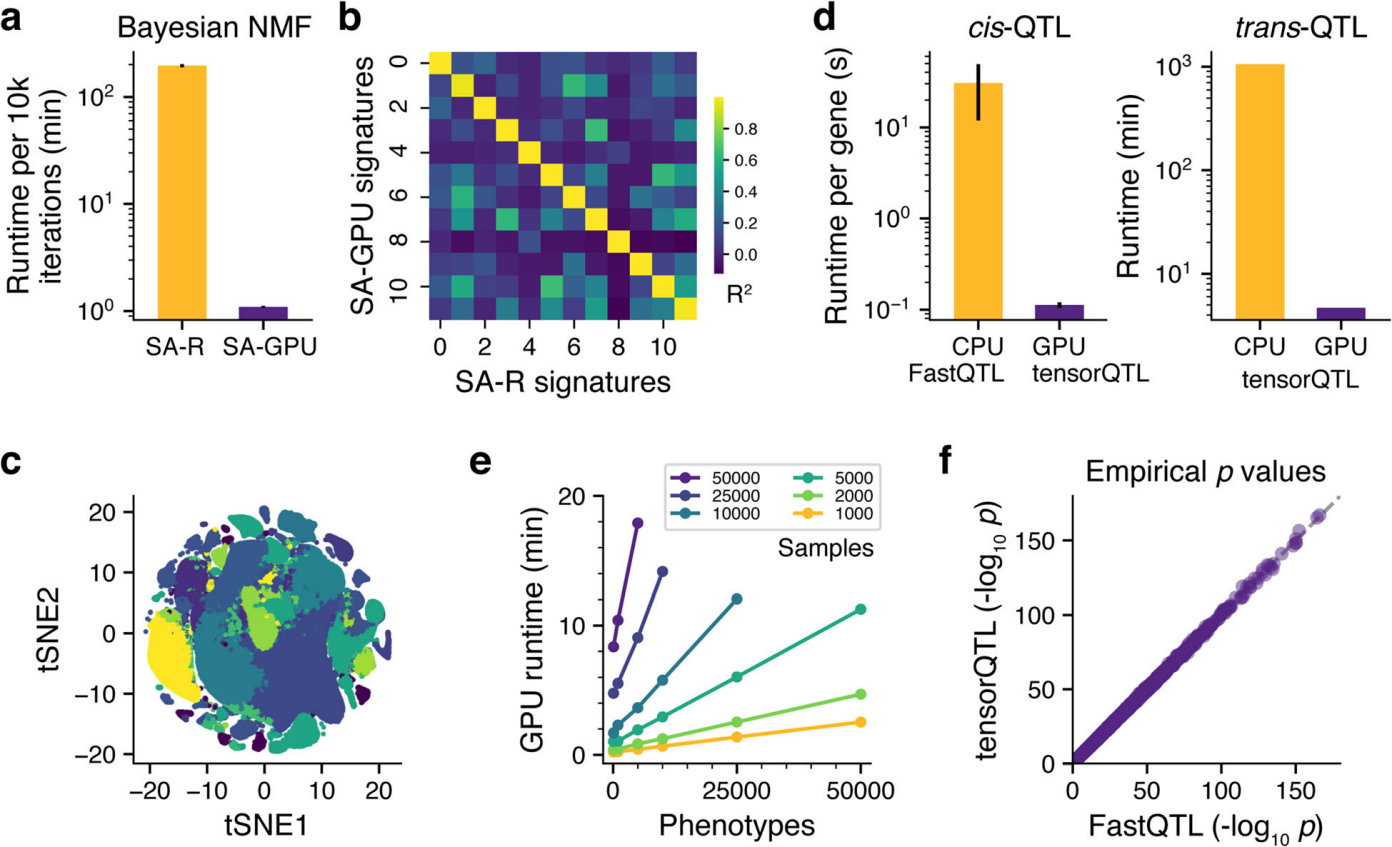
Performance of GPU implementations for QTL mapping and signature analysis. **a** Average runtime to compute 10,000 iterations of Bayesian NMF using SignatureAnalyzer (SA) in R (gold) and SignatureAnalyzer-GPU (SA-GPU; purple). **b** Correlation heat map of mutation signatures derived from the R and GPU implementations of SignatureAnalyzer using the same input mutation counts matrix. **c** *t*-distributed stochastic neighbor embedding (t-SNE) of 1 million embryonic mouse brain cells. Colors indicate clustering based on SA-GPU decomposition performed in ~ 15 min. **d** Comparison of runtimes for *cis*-QTL (FastQTL on CPU (gold) and tensorQTL on GPU (purple)) and *trans*-QTL (tensorQTL on CPU and GPU). **e** GPU runtime of tensorQTL for the indicated numbers of samples and phenotypes. **f** Empirical *cis*-eQTL *p* values from the V7 GTEx release replicated using tensorQTL. Error bars indicate standard deviation of the mean

To study the efficiency and benchmark the use of TensorFlow and PyTorch for large-scale genomic analyses on GPUs, we re-implemented methods for two commonly performed computational genomics tasks: (i) QTL mapping [9, 10] (which we call tensorQTL [11]) and Bayesian non-negative matrix factorization (NMF) [12] (named SignatureAnalyzer-GPU [13]). We executed the same scripts in identical environments (configured with and without a GPU) and also compared them to previous CPU-based implementations. As a baseline, we also benchmarked the performance of individual mathematical operations such as matrix multiplication, for which we observed up to ~ 1000-fold faster runtimes on a GPU vs. a single CPU core (Additional file 1: Figure S1 and Additional file 2). For SignatureAnalyzer-GPU (SA-GPU) [13], we used the mutation counts matrix generated by the Pan-Cancer Analysis of Whole Genomes (PCAWG) Consortium, which contains 2624 tumors represented by 1697 mutational features of somatic single-nucleotide variants as well as short insertions and deletions (defined based on their sequence contexts) [14]. Our PyTorch implementation ran approximately 200 times faster on a GPU than the current implementation of SignatureAnalyzer (SA) in R (run on a single CPU core), with mean times for 10,000 iterations of 1.09 min using SA-GPU vs. 194.8 min using SA (Fig. 1a). Using simulated data, we showed that SA-GPU scales linearly with the number of samples (Additional file 1: Figure S2A). When applied to previously published mutational signatures generated by SA [15], we found the results of the 2 methods were essentially identical, taking into account the stochastic nature of the underlying algorithm (mean *R*^2^ = 0.994, min *R*^2^ = 0.960; Fig. 1b). Additionally, we tested the performance of SA-GPU on multiple GPUs, a task that is easily achieved in PyTorch and enables, for example, faster hyperparameter optimization. For 20 decompositions using the same data as above, we found that performance scaled linearly with the number of GPUs and yielded equivalent results (Additional file 1: Figure S2B–C).

To further demonstrate the scalability of the Bayesian NMF to millions of data points, we used SA-GPU to identify the cell types and their associated transcriptional programs from single-cell RNA sequencing of 1 million mouse brain cells (SRA: SRP096558, Fig. 1c). The average time per SA-GPU run was 14.5 min (using a V100 Nvidia GPU; average over 10 runs) corresponding to an average of 6853 matrix updates per run. A similar analysis on a CPU would require > 2 days per run. Our analysis was able to identify 32 distinct transcriptional programs.

For tensorQTL [11] benchmarking, we generated random data representing up to 50,000 people, each with 10^7^ genotypes representing common variants. For each individual, we also simulated up to 50,000 phenotypes, resulting in 500 × 10^9^ all-against-all association tests (each calculated for up to 50,000 individuals). Our implementation of *cis*-QTL mapping with permutations to estimate the empirical false discovery rate was > 250 times faster than the current state-of-the-art implementation (FastQTL [10]; Fig. 1d). Likewise, *trans*-QTL mapping (i.e., 500 billion regressions) took less than 10 min, a ~ 200× increase in speed compared to running on a CPU (Fig. 1d and Additional file 1: Figure S3A). Our current implementation does not scale linearly as a function of samples (Additional file 1: Figure S3B) due to limitations in data transfer from the memory of the CPU to the GPU, rather than computational capacity; we leave this additional optimization for future work (Fig. 1e, Additional file 1: Figure S3B). We used data from the V6p and V7 releases of GTEx [16] generated using Matrix eQTL [9] and FastQTL [10], respectively, to demonstrate the reproducibility of our implementation (Fig. 1f and Additional file 1: Figure S3C).

In addition to the savings in computation time, implementation in TensorFlow or PyTorch also results in significant cost savings—at the time of writing, GPU compute time cost ~ $0.50–0.75/h on multiple cloud platforms compared to ~ $0.01–0.05/h for a CPU core. Thus, the same analyses were ~ 5–10-fold cheaper on GPUs.

## Conclusions

In summary, the implementation of many commonly used methods in genomics based on new GPU-compatible libraries can vastly increase runtime and reduce costs compared to CPU-based approaches. Indeed, by simply re-implementing current methods, we were able to achieve an order-of-magnitude higher increase in speed than may be achieved through sophisticated approximations for optimizing runtimes on CPUs [17, 18]. Our findings indicate that the scale of computations made possible with GPUs will enable investigation of previously unanswerable hypotheses involving more complex models, larger datasets, and more accurate empirical measurements. For example, our GPU implementation enables the computation of empirical *p* values for *trans*-QTL, which is cost-prohibitive on CPUs. Similarly, our results show that GPU-based approaches will enable scaling of single-cell analysis methods to millions of cells. Given the availability of libraries that obviate the need for specialized GPU programming, we anticipate a transition to GPU-based computing for a wide range of computational genomics methods.

## Methods

### tensorQTL

The core of tensorQTL is a reimplementation of FastQTL [10] in TensorFlow [7] and relies on pandas-plink (https://github.com/limix/pandas-plink) to efficiently read genotypes stored in PLINK [19] format into dask arrays [20].

The following QTL mapping modalities are implemented:

- *Cis*-QTL: nominal associations between all variant–phenotype pairs within a specified window (default ± 1 Mb) around the phenotype (transcription start site for genes), as implemented in FastQTL.
- *Cis*-QTL: beta-approximated empirical *p* values, based on permutations of each phenotype, as implemented in FastQTL.
- *Cis*-QTL: beta-approximated empirical *p* values for grouped phenotypes; for example, multiple splicing phenotypes for each gene, as implemented in FastQTL.
- Conditionally independent *cis*-QTL, following the stepwise regression approach described in [16].
- Interaction QTLs: nominal associations for a linear model that includes a genotype × interaction term.
- *Trans*-QTL: nominal associations between all variant–phenotype pairs. To reduce output size, only associations below a given *p* value threshold (default 1e−5) are stored.
- *Trans*-QTL: beta-approximated empirical *p* values for inverse-normal-transformed phenotypes, in which case the genome-wide associations with permutations of each phenotype are identical. To avoid potentially confounding *cis* effects, the computation is performed for each chromosome, using variants on all other chromosomes.

#### Benchmarking

To benchmark tensorQTL, we compared its *trans*-QTL mapping performance on a machine with and without an attached GPU, and *cis-*QTL mapping relative to the CPU-based FastQTL [10] (an optimized QTL mapper written in C++). For FastQTL, we computed the runtime per gene by specifying the gene and *cis*-window using the --include-phenotypes and --region options, respectively. The *cis*-mapping comparisons were performed using skeletal muscle data from the V6p release of GTEx [16]. To facilitate the comparison of GPU vs. CPU performance when mapping *trans*-QTLs across a wide range of sample sizes, we used randomly generated genotype, phenotype, and covariate matrices. All tensorQTL benchmarks were conducted on a virtual machine on Google Cloud Platform with 8 Intel Xeon CPU cores (2.30 GHz), 52 GB of memory, and an Nvidia Tesla P100 GPU. For CPU-based comparisons, computations were limited to a single core.

### SignatureAnalyzer-GPU

SA-GPU is a PyTorch reimplementation of SignatureAnalyzer [21], a method for the identification of somatic mutational signatures using Bayesian NMF [22]. SignatureAnalyzer was originally developed in R and is available for download at https://software.broadinstitute.org/cancer/cga/. Currently, SA-GPU requires the input data matrix and decomposition matrices (W and H) to fit into the GPU memory; however, since high-memory GPUs are readily available (e.g., Nvidia Tesla v100 has 16GB), we do not foresee this limiting its practical use. In case data sizes were to exceed this limit, the method is easily extensible to multiple GPUs using shared memory with built-in PyTorch methods.

SA-GPU can run a single Bayesian NMF or an array of decompositions in parallel, leveraging multiple GPUs. Users should specify a data likelihood function (Poisson or Gaussian) and either exponential or half-normal prior distributions on the elements of W and H, corresponding to L1 or L2 regularization, respectively.

#### Benchmarking

To benchmark the performance of SA-GPU, we compared SA-GPU with the previous implementation in R. We ran the R implementation using R 3.2.3 with the “Matrix” package for efficient matrix operations. All SA-GPU benchmarks were conducted on a virtual machine on Google Cloud Platform with 12 Intel Xeon CPU cores (2.30GHz), 20 GB of memory, and a Nvidia Tesla V100 GPU. For CPU-based comparisons, a single core was used.

## Supplementary information

**Additional file 1:** **Figure S1.** Performance of matrix multiplication on a single CPU core (2.30GHz Intel Xeon) vs. a GPU (Nvidia Tesla P100), using NumPy (compiled with OpenBLAS) and PyTorch. Runtimes were measured for multiplication of two random (uniform ~U[0,1]) square matrices (in 32-bit floating point) with the indicated dimensions. For the ‘PyTorch GPU’ runtimes, only the matrix multiplication itself was timed. For the ‘PyTorch GPU w/ copy’ runtimes, the copy of the two input matrices from CPU to GPU memory was included in the timing. Runtimes are shown as the median and median absolute deviation of 15 iterations each. **Figure S2.** Performance scaling of SignatureAnalyzer-GPU. **(a)** SignatureAnalyzer-GPU runtime scales linearly as a function of the number of samples. **(b)** Cumulative runtime for 20 runs of SignatureAnalyzer-GPU on a virtual machine configured with one or two GPUs (Nvidia Tesla V100). **(c)** Average number of signatures detected with one or two GPUs, indicating that the results are equivalent. The PCAWG mutation counts matrix was used for all comparisons. Error bars: standard deviation. **Figure S3.** GPU performance scaling of tensorQTL. **(a)** GPU-to-CPU runtime ratio for tensorQTL, across the indicated phenotype and sample sizes, for 10^7^ common variants. The ratio is non-constant due to data load and CPU-to-GPU memory input/output times (“i/o”) that are more limiting for large sample sizes (number of individuals). **(b)** CPU runtime of tensorQTL for the indicated range of sample and phenotype sizes (left panel). CPU runtimes scale linearly, demonstrated by the collapse of the compute time when measured as a function of number of operations (approximated as phenotypes × samples × variants; middle panel), whereas GPU runtimes do not show this collapse for large sample sizes due to i/o limitations (right panel). **(c)** Nominal significant *trans*-eQTL *p* values from the V6p GTEx release replicated using tensorQTL.

**Additional file 2.** Benchmarking code from Additional file 1: Figure S1

**Additional file 3.** Review history.

## Data Availability

All software is available on GitHub and implemented in Python using open-source libraries. tensorQTL is released under the open-source BSD 3-Clause License and is available at https://github.com/broadinstitute/tensorQTL [11]. SignatureAnalyzer-GPU is released under the open-source BSD 3-Clause License and is available at https://github.com/broadinstitute/SignatureAnalyzer-GPU [13].

## References

[1] Bycroft C, Freeman C, Petkova D, Band G, Elliott LT, Sharp K (2018). The UK Biobank resource with deep phenotyping and genomic data. Nature.

[2] McArt DG, Bankhead P, Dunne PD, Salto-Tellez M, Hamilton P, Zhang S-D (2013). cudaMap: a GPU accelerated program for gene expression connectivity mapping. BMC Bioinformatics.

[3] Mejía-Roa E, Tabas-Madrid D, Setoain J, García C, Tirado F, Pascual-Montano A (2015). NMF-mGPU: non-negative matrix factorization on multi-GPU systems. BMC Bioinformatics.

[4] Schatz MC, Trapnell C, Delcher AL, Varshney A (2007). High-throughput sequence alignment using graphics processing units. BMC Bioinformatics.

[5] Nobile MS, Cazzaniga P, Tangherloni A, Besozzi D (2016). Graphics processing units in bioinformatics, computational biology and systems biology. Brief Bioinform.

[6] Angermueller C, Pärnamaa T, Parts L, Stegle O (2016). Deep learning for computational biology. Mol Syst Biol.

[7] Abadi M, Agarwal A, Barham P, Brevdo E, Chen Z, Citro C (2016). TensorFlow: large-scale machine learning on heterogeneous distributed systems.

[8] Paszke A, Gross S, Chintala S, Chanan G, Yang E, DeVito Z (2017). Automatic differentiation in PyTorch.

[9] Shabalin AA (2012). Matrix eQTL: ultra fast eQTL analysis via large matrix operations. Bioinformatics.

[10] Ongen H, Buil A, Brown AA, Dermitzakis ET, Delaneau O (2016). Fast and efficient QTL mapper for thousands of molecular phenotypes. Bioinformatics.

[11] Aguet F, Taylor-Weiner A (2019). tensorqtl. GitHub.

[12] Kim J, Mouw KW, Polak P, Braunstein LZ, Kamburov A, Kwiatkowski DJ (2016). Somatic ERCC2 mutations are associated with a distinct genomic signature in urothelial tumors. Nat Genet.

[13] Taylor-Weiner A, Aguet F. SignatureAnalyzer-GPU. Github. 2019. https://github.com/broadinstitute/SignatureAnalyzer-GPU/. Accessed 15 Aug 2019.

[14] Alexandrov L, Kim J, Haradhvala NJ, Huang MN, Ng AWT, Boot A, et al. The repertoire of mutational signatures in human cancer. bioRxiv. 2018:322859 Available from: https://www.biorxiv.org/content/early/2018/05/15/322859. [cited 2018 Oct 1].

[15] Haradhvala NJ, Kim J, Maruvka YE, Polak P, Rosebrock D, Livitz D (2018). Distinct mutational signatures characterize concurrent loss of polymerase proofreading and mismatch repair. Nat Commun.

[16] GTEx Consortium (2017). Genetic effects on gene expression across human tissues. Nature.

[17] Loh P-R, Kichaev G, Gazal S, Schoech AP, Price AL (2018). Mixed-model association for biobank-scale datasets. Nat Genet.

[18] Zhou W, Nielsen JB, Fritsche LG, Dey R, Gabrielsen ME, Wolford BN (2018). Efficiently controlling for case-control imbalance and sample relatedness in large-scale genetic association studies. Nat Genet.

[19] Chang CC, Chow CC, Tellier LC, Vattikuti S, Purcell SM, Lee JJ (2015). Second-generation PLINK: rising to the challenge of larger and richer datasets. Gigascience.

[20] Rocklin M. Dask: parallel computation with blocked algorithms and task scheduling. Proc 14th Python Sci Conf. 2015:126–32 Available from: https://conference.scipy.org/proceedings/scipy2015/matthew_rocklin.html. [cited 2019 May 20].

[21] Kim J, Mouw KW, Polak P, Braunstein LZ, Kamburov A, Kwiatkowski DJ (2016). Somatic ERCC2 mutations are associated with a distinct genomic signature in urothelial tumors. Nat Genet.

[22] Tan V. Y. F., Fevotte C. (2013). Automatic Relevance Determination in Nonnegative Matrix Factorization with the /spl beta/-Divergence. IEEE Transactions on Pattern Analysis and Machine Intelligence.

