# Supplementary material for: Scaling computational genomics to millions of individuals with GPUs
Source: Genome Biol. 2019 Nov 1;20:228. doi: 10.1186/s13059-019-1836-7 (PMC6823959; doi:10.1186/s13059-019-1836-7)
Supplement: Supplementary file 1 — Additional file 1: Figure S1. Performance of matrix multiplication on a single CPU core (2.30GHz Intel Xeon) vs. a GPU (Nvidia Tesla P100), using NumPy (compiled with OpenBLAS) and PyTorch. Runtimes were measured for multiplication of two random (uniform ~U[0,1]) square matrices (in 32-bit floating point) with the indicated dimensions. For the ‘PyTorch GPU’ runtimes, only the matrix multiplication itself was timed. For the ‘PyTorch GPU w/ copy’ runtimes, the copy of the two input matrices from CPU to GPU memory was included in the timing. Runtimes are shown as the median and median absolute deviation of 15 iterations each. Figure S2. Performance scaling of SignatureAnalyzer-GPU. (a) SignatureAnalyzer-GPU runtime scales linearly as a function of the number of samples. (b) Cumulative runtime for 20 runs of SignatureAnalyzer-GPU on a virtual machine configured with one or two GPUs (Nvidia Tesla V100). (c) Average number of signatures detected with one or two GPUs, indicating that the results are equivalent. The PCAWG mutation counts matrix was used for all comparisons. Error bars: standard deviation. Figure S3. GPU performance scaling of tensorQTL. (a) GPU-to-CPU runtime ratio for tensorQTL, across the indicated phenotype and sample sizes, for 107 common variants. The ratio is non-constant due to data load and CPU-to-GPU memory input/output times (“i/o”) that are more limiting for large sample sizes (number of individuals). (b) CPU runtime of tensorQTL for the indicated range of sample and phenotype sizes (left panel). CPU runtimes scale linearly, demonstrated by the collapse of the compute time when measured as a function of number of operations (approximated as phenotypes × samples × variants; middle panel), whereas GPU runtimes do not show this collapse for large sample sizes due to i/o limitations (right panel). (c) Nominal significant trans-eQTL p values from the V6p GTEx release replicated using tensorQTL. [file 13059_2019_1836_MOESM1_ESM.pdf]

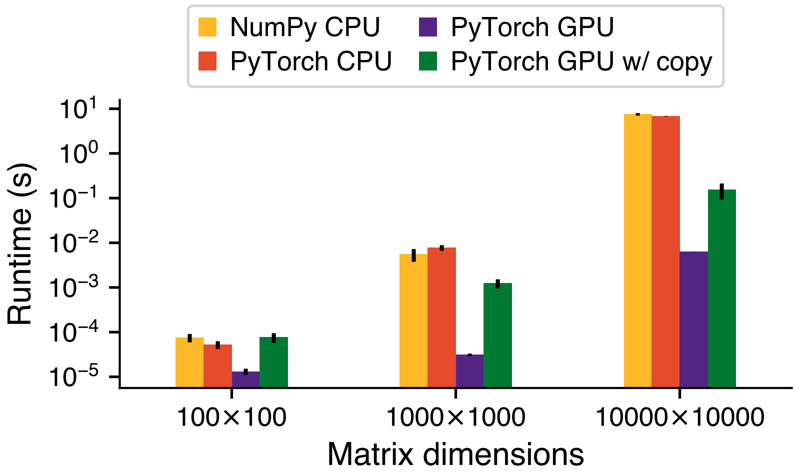

Fig. S1. Performance of matrix multiplication on a single CPU core (2.30GHz Intel Xeon) vs. a GPU (Nvidia Tesla P100), using NumPy (compiled with OpenBLAS) and PyTorch. Runtimes were measured for multiplication of two random (uniform ~U[0,1]) square matrices (in 32-bit floating point) with the indicated dimensions. For the 'PyTorch GPU' runtimes, only the matrix multiplication itself was timed. For the 'PyTorch GPU w/ copy' runtimes, the copy of the two input matrices from CPU to GPU memory was included in the timing. Runtimes are shown as the median and median absolute deviation of 15 iterations each.

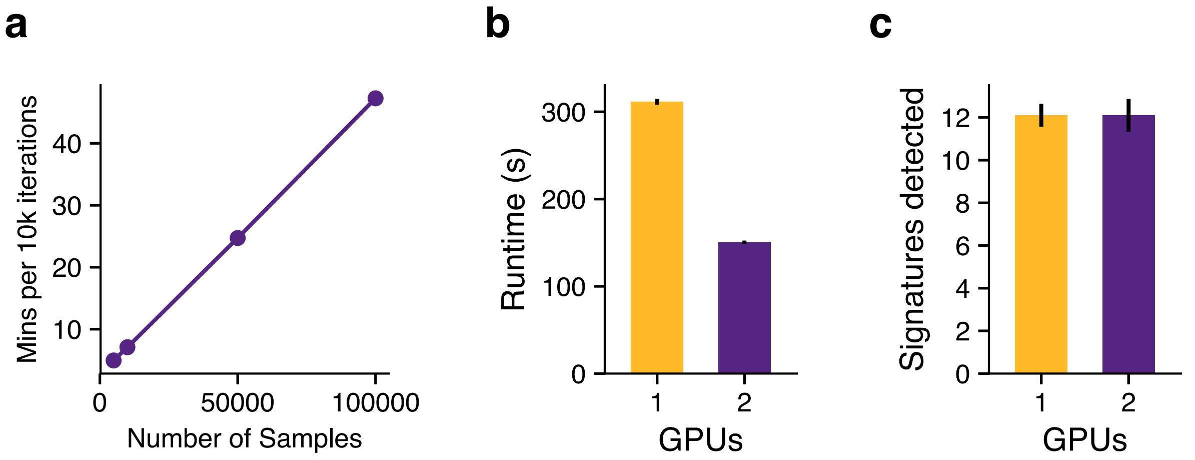

Fig. S2. Performance scaling of SignatureAnalyzer-GPU. **(a)** SignatureAnalyzer-GPU runtime scales linearly as a function of the number of samples. **(b)** Cumulative runtime for 20 runs of SignatureAnalyzer-GPU on a virtual machine configured with one or two GPUs (Nvidia Tesla V100). **(c)** Average number of signatures detected with one or two GPUs, indicating that the results are equivalent. The PCAWG mutation counts matrix was used for all comparisons. Error bars: standard deviation.

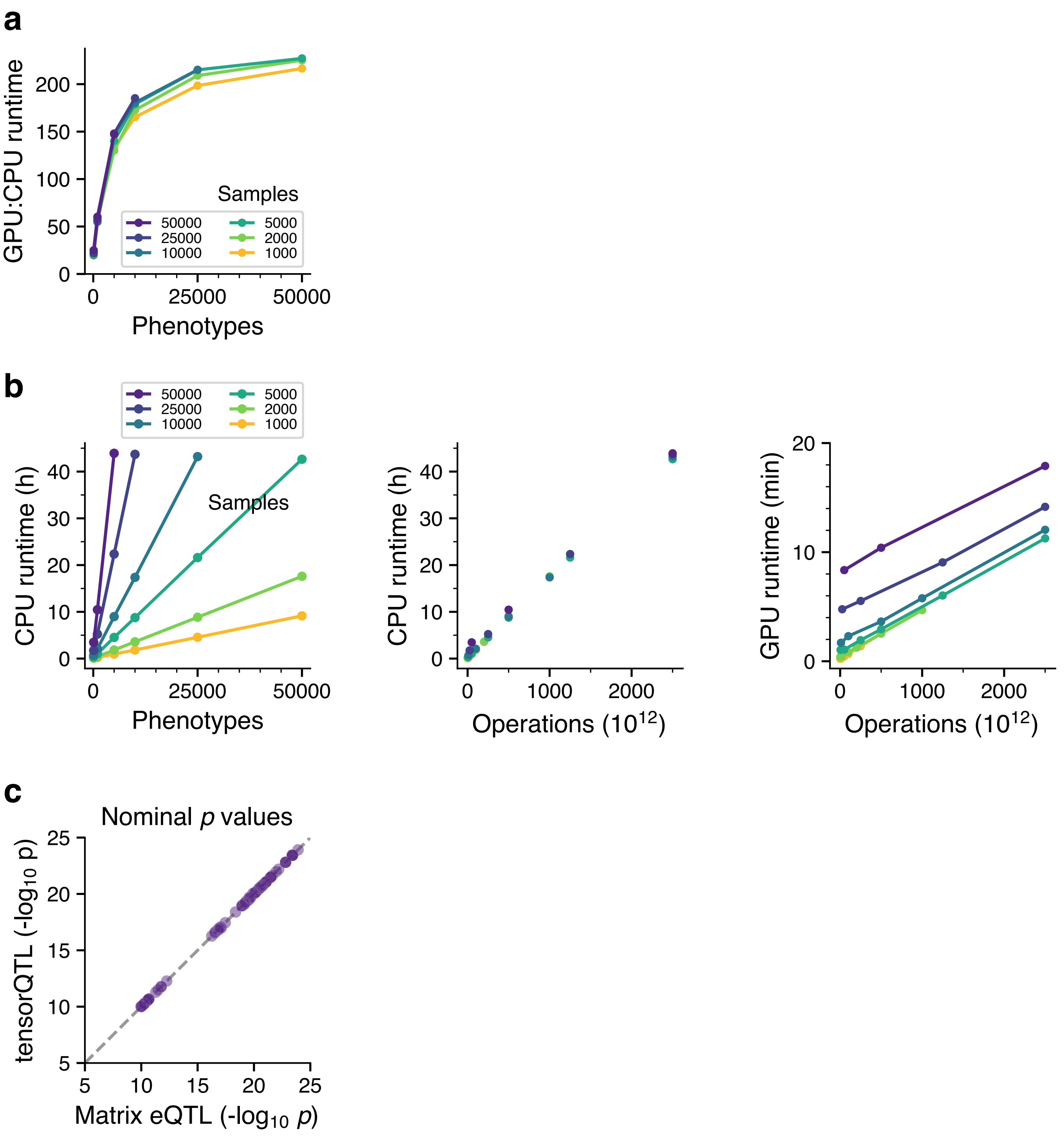

Fig. S3. GPU performance scaling of tensorQTL. **(a)** GPU-to-CPU runtime ratio for tensorQTL, across the indicated phenotype and sample sizes, for 10^7 common variants. The ratio is non-constant due to data load and CPU-to-GPU memory input/output times (“i/o”) that are more limiting for large sample sizes (number of individuals). **(b)** CPU runtime of tensorQTL for the indicated range of sample and phenotype sizes (left panel). CPU runtimes scale linearly, demonstrated by the collapse of the compute time when measured as a function of number of operations (approximated as phenotypes x samples x variants; middle panel), whereas GPU runtimes do not show this collapse for large sample sizes due to i/o limitations (right panel). **(c)** Nominal significant *trans*-eQTL *p* values from the V6p GTEx release replicated using tensorQTL.
