# Supplementary material for: Scaling computational genomics to millions of individuals with GPUs
Source: Genome Biol. 2019 Nov 1;20:228. doi: 10.1186/s13059-019-1836-7 (PMC6823959; doi:10.1186/s13059-019-1836-7)
Supplement: Supplementary file 3 — Additional file 3. Review history. [file 13059_2019_1836_MOESM3_ESM.docx]

Review History

**First round of review**

**Reviewer 1**

**Are you able to assess all statistics in the manuscript, including the appropriateness of statistical tests used?**

There are no statistics in the manuscript.

**Comments to author:**

The manuscript discusses the parallelization of two applications (called "TensorQTL" and "SignatureAnalyzer-GPU") on a GPU. Parallelization of computational genomics applications on GPUs is well-known for over a decade and has been studied extensively before. Unfortunately, the paper fails at explaining the utilized parallelization methodology; e.g. what are the challenges and  limitations in this context? The utilized parallelization method appears to be trivial and is simply based on library calls. However, it remains unclear what happens in situations where required algorithms are not included in the library or when data exceeds the limited memory capacities of GPUs. As a matter of fact, these points are key for achieving scalability for large-scale datasets. 

Reported benchmark results seem to be based on unoptimized sequential CPU programs; e.g. R programs. Thus, it remains unclear how significant the reported speedups are in comparison to optimized CPU (or GPU) programs. This would be especially important for large-scale datasets that exceed GPU memory.

**Reviewer 2**

**Are you able to assess all statistics in the manuscript, including the appropriateness of statistical tests used?**

There are no statistics in the manuscript.

**Comments to author:**

The short report by Taylor-Weiner et al. presents very promising results on the application of machine learning libraries, re-implemented on GPUs, for the analysis of large-scale genomics data.
GPUs are currently gaining a strong attention by the scientific community (see, for instance, the yearly workshop HiCOMB - IEEE International Workshop on High Performance Computational Biology), thanks to their capability in accelerating typical computational biology tasks that would be prohibitive on standard CPUs.
This short report holds the potential to make a real breakthrough in the analysis of genomic data of millions of samples.

I recommend the acceptance of the manuscript for publication, provided that the following minor revisions are taken into consideration by the authors.

- As this work is expected to be of interest for a wide range of readers interested in the use of both GPUs and deep learning methods in life science applications, I suggest citing the following papers:
1) doi:10.1093/bib/bbw058 (review on GPU-powered tools in bioinformatics, computational biology and systems biology)
2) doi:10.15252/msb.20156651 (review on machine/deep learning in computational biology)

- Page 2, line 8: graphical processing unit -> graphics processing unit

- Page 2, lines 44-45, and page 3, lines 5-8: the authors say they used in silico data to test the GPU-powered tools. Please provide more details (or a bibliographic reference) to explain how these data were generated.

- Page 2, line 57: please explain what "n=10" stands for.

- Page 3, line 12: 250x times -> remove x

- Bibliographic references 5 and 6 are not complete.

- Caption of Figure 1, panel a: reference to purple/gold colors is mixed up.

**Authors Response**

**Point-by-point responses to the reviewers’ comments:**

Point-by-point response

Reviewer #1:

The manuscript discusses the parallelization of two applications (called "TensorQTL" and "SignatureAnalyzer-GPU") on a GPU. Parallelization of computational genomics applications on GPUs is well-known for over a decade and has been studied extensively before.

*We agree with the reviewer that the advantages of GPUs for parallelization are well known. However, they are not widely utilized in computational genomics largely due to the complexity of writing and running GPU compatible software. For example, there has been a CUDA C++ library, with bindings in other languages, for many years but no bioinformatics tools that leverage GPUs are widely used. The likely reasons for this were: (i) the lack of ability to write the code once and then run it on either CPUs or GPUs (i.e. lack of portability of the code); and (ii) the limited availability of GPUs. Driven by methods developed for deep learning these limitations have largely disappeared. For example the PyTorch and TensorFlow libraries can use both CPUs and GPUs and are trivial to install and run. In addition, GPUs have become widely available on cloud platforms where they can be rented by the minute. Therefore, our manuscript aimed to communicate that GPU programming for bioinformatics methods is now greatly facilitated by general purpose libraries developed for deep learning.*

Unfortunately, the paper fails at explaining the utilized parallelization methodology; e.g. what are the challenges and limitations in this context? “The utilized parallelization method appears to be trivial and is simply based on library calls. However, it remains unclear what happens in situations where required algorithms are not included in the library “

*We thank the reviewer for this question that invoked us to provide further clarification. It is important to distinguish a library call from the methods that we present. Our methods are complete re-implementations of QTL mapping and regularized NMF that use general purpose mathematical operations (e.g. matrix multiplication) in TensorFlow and PyTorch. As such, our methods are not included in these libraries but rather were constructed using building blocks available within these frameworks. In order to leverage the TensorFlow/PyTorch GPU/CPU libraries, bioinformatic tools will need to be reimplemented using the operations provided in these libraries, as we did for the QTL and NMF methods. One of the messages of the manuscript is that it is now very easy to do so and we encourage the computational biology community to adopt this approach.*

“or when data exceeds the limited memory capacities of GPUs. As a matter of fact, these points are key for achieving scalability for large-scale datasets. “

*We thank the reviewer for highlighting this ambiguity and have added text to clarify how data that exceed GPU memory size is easily handled using PyTorch or TensorFlow. For example, tensorQTL performs QTL-mapping of 10^7 genotypes with 50k phenotypes which would require roughly 16TB of memory for the p-values alone. We handle this amount of calculation by storing the phenotype data on the GPU, streaming chunks of the genotype data to be analyzed, and storing only significant p-values. This batching and streaming is handled by the Tensorflow framework and did not require special development from us. Many genomic methods can benefit from this built-in batching and streaming capabilities since gene- or region-level analyses are typically inherently parallelizable.*

Reported benchmark results seem to be based on unoptimized sequential CPU programs; e.g. R programs. Thus, it remains unclear how significant the reported speedups are in comparison to optimized CPU (or GPU) programs. This would be especially important for large-scale datasets that exceed GPU memory.

*The performance improvements achievable with GPUs are well known with speed ups ranging from 10- to 1,000-fold across a range of applications (see e.g. Nobile et al. 2017 doi: 10.1093/bib/bbw058). Our own benchmarks were based on current implementations that are considered state-of-the-art in genomics and are widely used. We have clarified in the manuscript that FastQTL is implemented in C++ and is optimized. Similarly, Signature Analyzer in R uses the “Matrix” R package which calls BLAS and LAPACK libraries which are highly optimized for matrix multiplication. Therefore, the speed benefit that we observe is a direct consequence of the GPU capabilities (thousands of small cores) and not due to inefficient implementation on CPUs. For example, when we run the same TensorQTL code for trans-QTLs on CPUs vs GPUs we observe a ~200x speed-up which is comparable to the speed-up observed for cis eQTLs obtained from FastQTL on CPUs vs. TensorQTL on GPUs.*

Reviewer #2: The short report by Taylor-Weiner et al. presents very promising results on the application of machine learning libraries, re-implemented on GPUs, for the analysis of large-scale genomics data.

GPUs are currently gaining a strong attention by the scientific community (see, for instance, the yearly workshop HiCOMB - IEEE International Workshop on High Performance Computational Biology), thanks to their capability in accelerating typical computational biology tasks that would be prohibitive on standard CPUs.

This short report holds the potential to make a real breakthrough in the analysis of genomic data of millions of samples.

I recommend the acceptance of the manuscript for publication, provided that the following minor revisions are taken into consideration by the authors.

- As this work is expected to be of interest for a wide range of readers interested in the use of both GPUs and deep learning methods in life science applications, I suggest citing the following papers:

1) doi:10.1093/bib/bbw058 (review on GPU-powered tools in bioinformatics, computational biology and systems biology)

2) doi:10.15252/msb.20156651 (review on machine/deep learning in computational biology)

*We thank the reviewer for this suggestion. We have now cited these papers.*

- Page 2, line 8: graphical processing unit

-> graphics processing unit

*We have fixed this typo.*

- Page 2, lines 44-45, and page 3, lines 5-8: the authors say they used in silico data to test the GPU-powered tools. Please provide more details (or a bibliographic reference) to explain how these data were generated.

*We thank the reviewer for this. We have clarified how benchmarking was performed for both methods, and specified where simulated data was used to facilitate comparisons across wide ranges of sample sizes.*

- Page 2, line 57: please explain what "n=10" stands for.

*We have clarified that n=10 means 10 NMF runs.*

- Page 3, line 12: 250x times -> remove x

*Thank you. We removed the ‘x’.*

- Bibliographic references 5 and 6 are not complete.

*We have completed the references.*

- Caption of Figure 1, panel a: reference to purple/gold colors is mixed up.

*We thank the reviewer for highlighting this mistake. We have now fixed the colors.*

**Second round of review**

**Reviewer 1**

The manuscript presents some speedup results for parallelizing two applications (called "TensorQTL" and "SignatureAnalyzer-GPU") on a GPU. While the speedups appear to be high, I have a few concerns:

- Parallelization methodology: The parallelization methodology is not described. The authors claim that their methods are "complete re-implementations" that use mathematical operations in TF and PyTorch but have fail to include any details in the methodology section. Thus, it remains unclear what the challenges and limitations of their approach are. Especially, it remains unclear what happens in scenarios where required building blocks are not included in the utilized library. Furthermore, calling libraries to develop GPU-based applications is well-known and there are a number of other options, such as RAPIDS (https://github.com/rapidsai/cuml, https://github.com/rapidsai/dask-cuml) or directive-based performance-portable parallel programming models like OpenACC. Overall, the methods section is insufficient.

- Performance evaluation: The authors claim that their implementations are comapred to optimized CPU implementations. However, the reported speedups of 200x-300x clearly exceed the differences in peak performance of modern CPU/GPU hardware (the difference in their peak performance (or main memory bandwidth) is only around 10x). Thus, it remains unclear why a speedup is achieved that is orders-of-magnitude larger than the actual performance difference of the utilized devices. A description of the scaling of the CPUs with varying number of threads and vectorization efficiency would need to be included to better understand this behaviour.  

- The authors claim that they can leverage multiple GPUs but do not inlcude any performance results about GPU scaling.

- The hardware setup is not properly described. In particular, specifications of utilized CPUs, GPUs, and cloud platforms are not given.

In summary, speeding up bioinformatics applications on GPUs is well-known and I am not convinced that the paper advances the state-of-the-art in this area.

**Authors Response**

**Point-by-point responses to the reviewers’ comments:**

Reviewer #1: 

1) COMMENT: 

The manuscript presents some speedup results for parallelizing two applications (called "TensorQTL" and "SignatureAnalyzer-GPU") on a GPU. While the speedups appear to be high, I have a few concerns: 

- Parallelization methodology: The parallelization methodology is not described. The authors claim that their methods are "complete re-implementations" that use mathematical operations in TF and PyTorch but have fail to include any details in the methodology section. Thus, it remains unclear what the challenges and limitations of their approach are. Especially, it remains unclear what happens in scenarios where required building blocks are not included in the utilized library. Furthermore, calling libraries to develop GPU-based applications is well-known and there are a number of other options, such as RAPIDS (https://github.com/rapidsai/cuml, https://github.com/rapidsai/dask-cuml) or directive-based performance-portable parallel programming models like OpenACC. Overall, the methods section is insufficient. 

 *RESPONSE: 

We thank the reviewer for their comments, but believe there is a misunderstanding about the nature of the GPU-based libraries we describe, and the implicit parallelization they enable. We specifically used TensorFlow and PyTorch in our implementations because these libraries are, to the best of our knowledge, the most feature-complete, and implement many if not all of the mathematical primitives found in CPU-based general-purpose libraries such as NumPy. Parallelization in these libraries is implicit (i.e. not needed to be specified by the developer), in the sense that mathematical primitives executed on the GPU utilize the thousands of cores available on these GPU chips. Moreover, code written using NumPy (which is the most widely-used Python library for scientific computing) can easily be ported to TensorFlow or PyTorch, since many of the function names are similar or identical. 

PyTorch and TensorFlow are also by far the most widely-used GPU libraries reflected by adoption metrics such as ‘stars’ on GitHub, which are a measure of the number of users actively involved in or following open-source projects. TensorFlow is the fourth-most popular repository on GitHub by this measure (with ~132,000 stars; https://github.com/search?q=stars%3A%3E0&s=stars&type=Repositories), and PyTorch also has a high number of associated users, even compared to NumPy (~30,500 vs ~11,400 stars). 

To the best of our understanding, cuML is a collection of individual algorithms (listed at https://github.com/rapidsai/cuml), rather than a general-purpose library for scientific computing (this is also reflected by the lower number of users, ~600 stars on GitHub). Such specialized libraries, which don’t rely on highly optimized mathematical primitives (as implemented in PyTorch and TensorFlow), often result in significantly smaller performance improvements (<10x); this is also the case for OpenACC (see https://developer.nvidia.com/openacc). 

The reason we believe libraries like PyTorch and TensorFlow will have a significant impact in enabling wide-spread use of GPU-based computing is precisely because they are general-purpose libraries and are as easy to use as popular CPU-based libraries such as NumPy. As mentioned above, the developers do not need to decide on the parallelization methodology, since these decisions are optimized within PyTorch and TensorFlow. We thank the reviewer for this comment and have now emphasized this distinction in the text.* 

2) COMMENT: 
- Performance evaluation: The authors claim that their implementations are comapred to optimized CPU implementations. However, the reported speedups of 200x-300x clearly exceed the differences in peak performance of modern CPU/GPU hardware (the difference in their peak performance (or main memory bandwidth) is only around 10x). Thus, it remains unclear why a speedup is achieved that is orders-of-magnitude larger than the actual performance difference of the utilized devices. A description of the scaling of the CPUs with varying number of threads and vectorization efficiency would need to be included to better understand this behaviour. 
 *RESPONSE: 

We thank the reviewer for highlighting a need for further clarification of the performance increases that are achievable with GPUs vs. a single threaded CPU. Many of the bioinformatics methods and algorithms are limited by their computational needs rather by I/O (or memory bandwidth). We added a supplementary figure (the new Supplementary Fig. 1) to demonstrate the decrease in runtime on GPU, both with and without accounting for copying data to the GPU memory. Using matrix multiplication as an example, we demonstrate up to ~1000-fold decreases in runtime on a GPU (compared to a single CPU core). Even when memory copy operations are included, the runtime decrease remains close to ~100-fold (please note that this ~100-fold represents a lower bound, as practical applications will likely involve more than a single operation after transferring data to the GPU). For CPU-based benchmarks, we used NumPy compiled with OpenBLAS, which provides close to optimal performance on CPUs. WE used a single CPU core since most used methods do not utilize multithreading consistently and since costs on cloud computing are based on rented cores. While we acknowledge that relatively marginal improvements to CPU performance are possible (using, e.g., the Intel MKL library instead of OpenBLAS), our intention was to demonstrate out-of-the-box improvements that are close to optimal. A jupyter notebook with the code for these benchmarks is included as supplementary information.* 

3) COMMENT: 
- The authors claim that they can leverage multiple GPUs but do not inlcude any performance results about GPU scaling. 
 *RESPONSE: 
We thank the reviewer for pointing this out, and have now included additional panels in Supplementary Figure 2 to demonstrate this capability and show that performance scales linearly with the number of GPUs.* 

4) COMMENT: 
- The hardware setup is not properly described. In particular, specifications of utilized CPUs, GPUs, and cloud platforms are not given. 
 *RESPONSE: 
We thank the reviewer for highlighting this omission and have added descriptions of the hardware setups used to the methods section.* 

5) COMMENT: 
In summary, speeding up bioinformatics applications on GPUs is well-known and I am not convinced that the paper advances the state-of-the-art in this area. 

*RESPONSE: 
We thank the reviewer for this comment but respectfully disagree. While there are indeed examples of GPU-based bioinformatics methods (cited in the text, Ref. 2-6), these often rely on specialized implementations and are not widely used. To the best of our knowledge, there are currently no widely-used genomics methods implemented on GPUs, despite the urgent need for more computationally efficient methods (see for example any of the recently published GWAS methods, or methods for processing single cell data, which are all CPU-based and can take hours or even days to run on multi-core machines). Indeed, neither the dramatic GPU-based speedups enabled by machine-learning libraries such as PyTorch and TensorFlow, nor the fact that these are general-purpose libraries, appear to be known or leveraged in the computational biology community. We hope that our manuscript will make the community aware of these easy-to-use libraries and the innovations that they enable, and use our tools for the research and as templates for porting their own tools to GPUs.*

**Third round of review**

**Reviewer 2**

The authors have satisfactorily addressed all the issues raised in the previous round of review, I warmly suggest acceptance of the revised manuscript for publication.
